# A Holistic Approach to ZigBee Performance Enhancement for Home Automation Networks

**DOI:** 10.3390/s140814932

**Published:** 2014-08-14

**Authors:** August Betzler, Carles Gomez, Ilker Demirkol, Josep Paradells

**Affiliations:** 1 Department of Telematics Engineering, Universitat Politècnica de Catalunya, 08034 Barcelona, Spain; E-Mails: carlesgo@entel.upc.edu (C.G.); ilker.demirkol@entel.upc.edu (I.D.); josep.paradells@entel.upc.edu (J.P.); 2 Fundacio i2CAT, 08034 Barcelona, Spain

**Keywords:** ZigBee, home automation, wireless sensor networks, IEEE 802.15.4, performance improvement

## Abstract

Wireless home automation networks are gaining importance for smart homes. In this ambit, ZigBee networks play an important role. The ZigBee specification defines a default set of protocol stack parameters and mechanisms that is further refined by the ZigBee Home Automation application profile. In a holistic approach, we analyze how the network performance is affected with the tuning of parameters and mechanisms across multiple layers of the ZigBee protocol stack and investigate possible performance gains by implementing and testing alternative settings. The evaluations are carried out in a testbed of 57 TelosB motes. The results show that considerable performance improvements can be achieved by using alternative protocol stack configurations. From these results, we derive two improved protocol stack configurations for ZigBee wireless home automation networks that are validated in various network scenarios. In our experiments, these improved configurations yield a relative packet delivery ratio increase of up to 33.6%, a delay decrease of up to 66.6% and an improvement of the energy efficiency for battery powered devices of up to 48.7%, obtainable without incurring any overhead to the network.

## 1. Introduction

In our daily lives, wireless home automation networks (WHANs) promise an important role, as the devices in common use can easily be connected with each other to enhance user comfort and to allow efficient home management. The potential of such wireless devices increases further as the connection to the Internet becomes possible for them. WHANs present a strong alternative to wired networks for home automation systems, and they have been a target of several standardization efforts [1,2]. Among these efforts, ZigBee has been considered as a strong candidate to be adopted for WHANs [3]. The ZigBee protocol stack has been the subject of several performance studies [4–7], some of which focus on the use of ZigBee for WHANs [8,9]. Various detailed evaluations have been carried out for specific ZigBee layers [10–12]. On the other hand, ZigBee has been defined on the basis of components developed and widely studied by the research community. However, these components have been considered separately. In fact, a holistic evaluation of the ZigBee protocol stack, which is vital to observe the overall performance of the specification, is not publicly available in the literature.

The generic ZigBee specification [13] defines a set of protocol mechanisms and parameters, the latter with default and allowed values. Moreover, for specific application types, ZigBee defines application profiles. One of these is the ZigBee Home Automation (HA) profile [14], which includes specific settings for WHAN scenarios. A trivial and mainly the common way of configuring the network parameters and mechanisms in a deployment is to keep their default settings from the specifications. Hence, it is crucial to understand the potential performance gains that can be achieved by tuning the settings of the protocol stack. Therefore, an evaluation of the default and the allowed settings defined in the ZigBee specification and HA profile is needed.

In this paper, we study the end-to-end network performance of ZigBee WHANs following a holistic approach, which considers parameter settings and mechanism designs from different protocol stack layers. The goal behind these evaluations is to quantify possible performance improvements through the tuning of these settings and, also, to provide a future direction for the evolution of the ZigBee specifications. Performance evaluations are done: (i) for the default protocol stack configuration, which corresponds to using the default settings of the investigated parameters and mechanisms; (ii) for various alternative configurations that are also compliant with the ZigBee specifications; and (iii) for alternative settings that do not disrupt the ZigBee architecture, but are however not compliant with the specifications.

For the study, we run various application scenarios that have been derived according to the ZigBee HA profile and Internet Engineering Task Force (IETF) specifications for home automation [1]. Experiments are carried out on a testbed of 57 nodes, where different network conditions are defined and evaluated. Specifically, we define different network topologies and consider the effect of the transmit power setting in these. We also define different types of traffic generation models along with varying corresponding loads. Moreover, we partially randomize the source and destination nodes for a given traffic load to devise the average behavior of the evaluated approaches.

The investigated performance metrics are end-to-end packet delivery ratio (PDR), end-to-end delay and energy efficiency, which are three common and crucial network performance criteria for WHANs. These metrics are evaluated for various combinations of settings of key network parameters and mechanisms from different stack layers. Based on the findings from our experiments, we propose two new protocol stack configurations, namely the Recommended-Compliantconfiguration and the Recommended-Unrestricted configuration. The two recommended configurations achieve much higher end-to-end PDR, a lower end-to-end delay and a higher energy efficiency than the default ZigBee settings in our experiments. Numerically, the Recommended-Unrestricted settings yield up to a 33.6% relative PDR increase, up to a 66.6% delay decrease and up to a 48.7% energy efficiency improvement in comparison with the default ZigBee settings. These improvements are achieved without incurring any overhead to the network. In addition, we provide detailed insight into the factors within and across the analyzed ZigBee layers that have a significant influence on the performance. Note that the findings of this work are not limited to ZigBee networks and also provide useful guidelines for the design and performance improvement of other IEEE 802.15.4-based networks.

The rest of the paper is structured as follows. Related work is presented in Section 2. An overview of ZigBee is given in Section 3.1. The default configuration of the ZigBee protocol stack and the investigated network parameters and mechanisms are introduced in Section 3.2. In Section 4, the evaluation environment is described. In Section 5, the results of the evaluations are presented. Based on the results gained, the two improved configurations of the ZigBee protocol stack are proposed and evaluated in Section 6. In Section 7, the recommended settings are evaluated in alternative network scenarios. Section 8 concludes the paper.

## 2. Related Work

To our best knowledge, no performance analysis of the mechanisms and parameters of the complete ZigBee communication protocol stack has been provided. Publicly available studies focus on a single ZigBee layer or on testing the performance of default ZigBee.

Within these studies, only a few use a real testbed environment to carry out their evaluations, while the majority backs its results on simulations. However, simulations provide a lower degree of accuracy in some aspects that are relevant to the outcome of experiments, such as the radio signal propagation in a real environment, the interference observed and the actual delay incurred by the devices in the network.

From the few papers that base their evaluations on a real testbed, ZigBee-capable hardware is designed and tested in an indoor environment using 51 nodes in [4]. The focus of the evaluation lies in determining long-term loss rates and transmission throughput. Contrary to our investigation, only the capabilities of the default ZigBee protocol stack configuration are analyzed. At a smaller scale, a WHAN of four ZigBee devices is used in [8] to demonstrate that a specific WHAN architecture defined by the authors is stable and that the co-existence with IEEE 802.11 is feasible. A more realistic approach with several indoor and outdoor WHAN testbed setups that include approximately 10 real nodes per scenario is studied in [9]. Transmission failure rates and radio transmission ranges are evaluated depending on the positioning of the nodes within the scenarios. Amongst the scenarios evaluated, there are ones specifically designed to represent WHANs. However, the presented analysis does not focus on the influence of different protocol stack layer configurations on the network performance, only giving results about PDRs achieved by a single configuration in each of the scenarios. The small size of this WHAN testbed and the simple performance analysis do not give insights into how the ZigBee stack performs with a multitude of devices and a larger network extent, or for different ZigBee settings.

There exist evaluations of ZigBee networks that rely on simulations, focusing mainly on a specific network mechanism or parameter and on its optimization to achieve a better network performance. The work presented in [5] analyzes the performance of two basic routing methods for ZigBee networks and presents the performance improvement of a new routing protocol with a simulator. The sole focus of improving the routing protocol independently from other layers and a simulation-based approach deliver insufficient information about the realistic ZigBee stack performance in WHANs. Furthermore, by using a simulation environment, several additions to the default ZigBee protocol stack are proposed in [6] to enable a reliable data transmission control method for situations where data congestion problems are expected. The authors of this work demonstrate the contribution of their proposal with simulated networks of up to 100 nodes and compare the achieved performance to the one obtained by the default ZigBee protocol. However, the transmission of large amounts of data to a sink node is not characteristic of a WHAN scenario, where small amounts of data are exchanged and the one-to-one traffic pattern must also be considered [1].

Kohvakka *et al.* mathematically analyze MAC layer mechanisms of a large scale IEEE 802.15.4 network that performs ZigBee network operations inside a cluster-tree topology and establish run-time operation models of the nodes [7]. The models and results are confirmed via simulations. The two performance metrics evaluated in that work are power consumption and goodput. However, the large network size used for these evaluations of up to 1560 nodes exceeds the realistic size of a WHAN [15], and the use case of data gathering (*i.e.*, many-to-one traffic directed to a sink node) is not representative for a typical WHAN scenario.

Common alternatives to ZigBee for WHANs include Z-Wave [16], Bluetooth Low Energy (BLE) [17] and Wi-Fi (IEEE 802.11) [18]. An introduction of different technologies used in machine-to-machine (M2M) home automation environments is done by Starsinic [19]. The choice of a communication technology for a given WHAN environment will depend on the specific requirements of the latter. Among the alternative technologies for WHANs, a promising one is low-power Wi-Fi, given the widespread availability of Wi-Fi infrastructure in home scenarios. For example, Ostermaier *et al.* show that an energy-efficient connection of devices to the Internet of Things over devices that use low-power Wi-Fi is possible, even when using standards like HTTP and TCP/IP [20]. Wu *et al.* state that Wi-Fi technology is adequate to serve as the interface for home area networks to smart meters in smart grid environments [21]. While the market share of WHAN technologies, such as low-power Wi-Fi and also BLE, is growing steadily, ZigBee currently dominates the WHAN market [22]. Table 1 [23] shows a comparison of the key features, such as Bitrates or message (msg.) sizes used by several WHAN low-power technologies.

## 3. ZigBee Protocol Stack Overview and Configuration

In this section, a short introduction to the ZigBee protocol stack is given. Afterwards, the default ZigBee protocol stack configuration and the alternative settings of parameters and mechanisms that are investigated are presented.

### 3.1. ZigBee Overview

The ZigBee stack consists of four layers: the physical (PHY), the medium access control (MAC), the network (NWK) and the application (APL) layers. These layers are described next with a special emphasis on the settings that we evaluate in this paper.

The PHY and MAC layers are adopted from the IEEE 802.15.4 specification, which defines mechanisms for, amongst others, the one-hop communication and channel access coordination amidst the nodes [24]. The IEEE 802.15.4 PHY layer is defined for three different radio frequency bands: 868 MHz, 915 MHz and 2.4 GHz. In the 2.4 GHz frequency band, which is available worldwide, orthogonal-quadrature phase shift keying (O-QPSK) is used to modulate the radio signal. The direct sequence spread spectrum (DSSS) is applied, increasing the robustness of data transmissions. With these settings, data rates of up to 250 kbit/s can be achieved. In the alternative frequency bands, which are used in Europe (868 MHz) and North America (915 MHz), binary phase shift keying (BPSK) is used in combination with DSSS, facilitating data rates of 20 kbit/s and 40 kbit/s, respectively.

IEEE 802.15.4 defines two node roles, namely full-function devices (FFDs) and reduced-function devices (RFDs). While an FFD can talk to any other device, RFDs can only communicate with FFDs. In addition, only FFDs take part in routing. The resources of an RFD, like memory and processing power, are normally more restricted than the ones of an FFD. RFDs apply duty cycling to reduce energy consumption.

A ZigBee network may operate in the beacon-enabled or beacon-less mode of IEEE 802.15.4, depending on the necessities of the application and the network topology. While in the beacon-enabled mode, channel access is organized by means of a superframe structure defined by beacons; in the beacon-less mode, nodes compete for the channel access without centralized coordination. According to the specification, in hierarchical (*i.e.*, tree) topologies any of these modes can be used; however, in mesh topologies, beacon-less mode is the only option. In the beacon-enabled mode, the time between two beacons is split into the contention access period (CAP), the contention-free period (CFP) and an inactive period. While in CFP, devices are assigned guaranteed time slots (GTSs), in CAP, as well as in the beacon-less mode, devices employ carrier sense multiple access with collision avoidance (CSMA/CA). In CSMA/CA, before the transmission of data is initiated, a random backoff is applied and the channel occupation is sensed. If the channel is found to be busy, the channel access procedure is repeated up to a certain number of times.

Independently of the operational mode, the IEEE 802.15.4 MAC layer provides optional one-hop reliability by using MAC layer acknowledgments (ACKs) to confirm the reception of packets. By default, MAC layer ACKs are enabled. If an ACK is not received after a specific duration (54 symbol durations), the delivery of the data frame is supposed to have failed. After an unsuccessful delivery, a retransmission is initiated, if the allowed retransmission count has not been reached for that data frame. This value is defined by the MAC layer parameter *macMaxFrameRetries* [24]. Once this many retries have been performed without receiving an ACK, the transmission of the data frame is considered to be unsuccessful. Possible values for the *macMaxFrameRetries* parameter are defined to be between zero and seven, with a default value of three. The analysis of different configurations of this parameter is important, since it is related to the one-hop reliability and the link break detection mechanism of the NWK layer.

The ZigBee NWK layer is responsible for, among other functions, discovering and maintaining routes between devices and relaying messages. It specifies a hierarchical routing scheme for the hierarchical (tree) topology and a peer-to-peer routing protocol based on the *Ad hoc* On-demand Distance Vector routing protocol (AODV) [25] for the mesh topology. While the hierarchical topology is mainly designed for many-to-one traffic, the mesh topology facilitates the communication between any two nodes of the network, *i.e*., it supports point-to-point traffic. In mesh topologies, the basic procedure of finding a route consists in disseminating Route Request (RREQ) messages across the network, parting from the source node, until the destination node is reached. The destination node then responds with a Route Reply (RREP) message that is propagated back to the source node over the discovered route. The route selection is based on the path cost calculation metric used by ZigBee, which computes a link cost *C*{*l*} for every link on a route and calculates the cost of a path as the summation of the costs of all of the links along the path. When multiple RREQ messages reach the destination, the route corresponding to the one with the minimum total path cost is selected. *C*{*l*} is defined to range between one and seven and is calculated as:

(1) $$C\{l\}=\{\begin{array}{ll} \min\left(7,\text{round}\left(\frac{1}{p_{l}^{4}}\right)\right) & (\text{default}),\\ 7 & (\text{optional}), \end{array}$$

where *p_l_* is the one hop link delivery ratio (LDR) [13]. The LDR of a link can be estimated from the link quality indication (LQI) calculated by the receiving node on the reception of a packet. The LQI may be obtained from measurements of the signal-to-noise ratio (SNR), the received signal strength indicator (RSSI) or a combination of both. The conversion of LQI to LDR is not specified in the specification and is up to the implementation. Setting the link cost to a fixed value, as in Equation (2), is also an option provided by the ZigBee specification. This option is equivalent to applying the hop count (HC) metric that chooses the shortest available route.

The APL layer is the top layer of the ZigBee protocol stack, which provides an optional end-to-end reliability mechanism to assure the successful delivery of data between two end nodes. End-to-end ACKs can be used to confirm the reception of the data at the destination node. The loss of a packet is assumed if after a certain amount of time, no application layer acknowledgment (APL ACK) has been received from the destination node. Then, a retransmission of the data packet is initiated. The duration of the time interval during which a response is expected is specified by the retransmission timeout (RTO) value. ZigBee uses blockwise acknowledgments, which are used to confirm the successful reception of several packets at once, similar to the cumulative acknowledgment method of Transmission Control Protocol (TCP) [26]. The recommended block size according to the ZigBee HA profile is one. With these settings, the end-to-end reliability mechanism in a ZigBee WHAN behaves like a simple stop-and-wait mechanism with positive ACKs.

### 3.2. Protocol Stack Configuration

**Figure 1.**
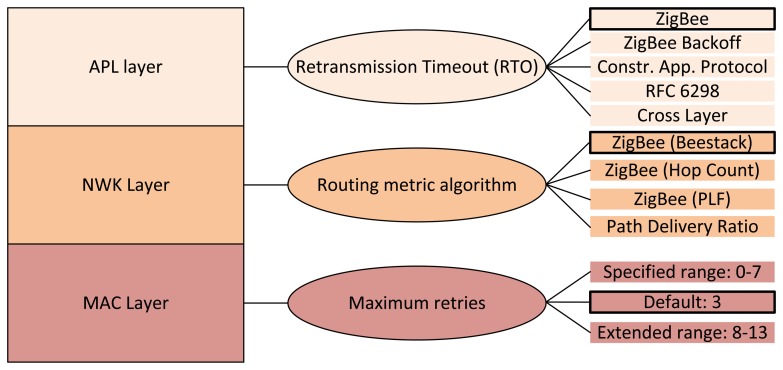
Simplified overview of the ZigBee communication protocol stack and the parameters/mechanisms that are evaluated from different layers and the default and alternative settings for these parameters/mechanisms. The default settings are highlighted with bolded boxes.

Because parameters and mechanisms defined for the considered ZigBee communication protocol stack layers are numerous, only a specific subset that is expected to have a high impact on the network performance is evaluated. Specifically, we investigate the effect of MAC retries from the MAC layer, the routing metric from the NWK layer and the RTO algorithm from the APL layer. Figure 1 gives an overview of the parameters and mechanisms considered in the evaluations. In addition, the default and the alternative settings are also shown in the figure.

#### 3.2.1. MAC Layer

In a previous work, the effect of allowing different maxima for the amount of MAC layer retries has proven to be significant for the performance of applications that involve bulk data transfers between two nodes, such as file transfer applications [27]. In this paper, the maximum amount of allowed MAC layer retransmissions defined by the *macMaxFrameRetries* parameter is analyzed with regard to its influence on the one-hop reliability and the overall network performance in the investigated WHAN scenarios. The results presented in Section 5.1 show that the one-hop reliability is heavily correlated to this setting and that such reliability is crucial for the performance of end-to-end communications. Apart from doing evaluations within the range defined in the ZigBee specification, *i.e.*, from zero to seven retransmissions, also the effect of using values from an extended range of up to 13 retries is analyzed, to see if one-hop reliability increases further and how the network performance changes. On the other hand, alternative backoff methods for accessing the channel have been tested in previous work, showing that the impact of this mechanism can be relevant for single end-to-end large file transfers [27]. However, for the application cases analyzed in this paper, the backoff methods were shown to have no relevant impact on network performance.

#### 3.2.2. NWK Layer

**Figure 2.**
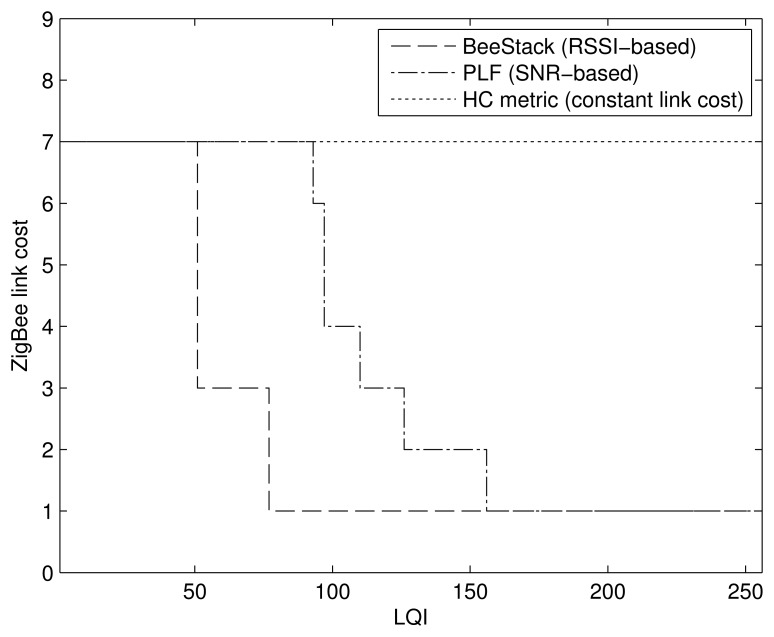
Mapping of link quality indication (LQI) to link cost values by the BeeStack [30], hop count (HC) and piecewise linear function (PLF) [31] routing metrics.

The devices in WHAN applications have the capability to communicate with any other device in the network [1,28], making the mesh topology the proper choice for the network topology. Thus, an important MAC layer setting in the evaluation carried out in this paper is the use of the beacon-less mode, since the ZigBee mesh network topology only supports such a mode, as described in Section 3.1. Most of the devices are FFDs, except for a few RFDs that do not participate in routing. From the mechanisms provided by the NWK layer for the mesh topology, the impact of the routing metric on network performance is evaluated. The default routing metric uses Equation (1) to calculate the link costs. Although an LDR value is required by Equation (1), that value may not be available to a ZigBee node. Alternatively, the ZigBee specification suggests using a mapping from LQI to a link cost, the details of which are not specified and are left to the implementation. A prevalent ZigBee implementation from Freescale, BeeStack [29], calculates the LQI based on the RSSI of a packet and converts it into a link cost, the mapping of which is shown in Figure 2.

The link cost mapping used in the BeeStack implementation is assumed as the default link cost setting for the evaluations of this paper, the performance of which is compared to the performance achieved with two other interpretations of the ZigBee metric. The first consists in using constant link costs resulting in the HC metric, *i.e.*, using Equation (2) as the link cost. The second is a piecewise linear function (PLF) mapping from LQI to LDR derived by the physical experiments in [32] and then applying Equation (1). The LQI value in this case is calculated from the correlation value provided by the radio transceiver as an SNR estimate [33]. The PHY layer implementation obtains the LQI by correlating the first eight symbols after the start-of-frame delimiter of a packet. The PLF mapping is also shown in Figure 2. Note that the BeeStack bases its LQI calculation on the RSSI of a packet, while the PLF metric uses the SNR estimate obtained by the correlation to determine the LQI value. The reader is encouraged to see the survey on radio link quality estimation by Baccour *et al.* for detailed information on these metrics and alternative ones [34].

As an alternative routing metric, the Path-DR metric [35] is included in the evaluations. Path-DR estimates the LDR of each link during a route discovery. In the evaluations, this estimation is also implemented based on the LQI to LDR mapping derived by Gomez *et al.* [32]. By multiplying the estimated LDR values at each link, an end-to-end PDR estimation is obtained when an RREQ packet reaches the destination node. The Path-DR metric selects the route with the highest estimated PDR. This metric is not ZigBee-compliant, since it does not apply the ZigBee path cost calculation algorithm. The reasons to include this metric in the evaluations are two-fold. First, it has been shown that this metric results in better network performance in an indoor scenario than other route selections metrics, such as HC and max-LQI [35]. Secondly, we consider a metric that is not ZigBee-compliant to be a benchmark for the ZigBee-compliant metrics.

#### 3.2.3. APL Layer

At the APL layer, the impact of the RTO algorithm on the network performance is evaluated. Since there is not one universal RTO value that always delivers an optimal performance, there is a need to use an RTO algorithm that adapts to the requirements of the application and the network conditions. In this context, the performances of four alternative RTO algorithms are evaluated and compared to the default one. An RTO algorithm can be split into two main components: the initial RTO assignment and the backoff method. The initial RTO value is used for the first transmission of a packet. If a loss is detected, *i.e.*, if the RTO timer expires and the packet needs to be retransmitted, a backoff mechanism can be applied, which increases the RTO value. In the following, a short introduction to the five RTO algorithms investigated is given. A summary of these five RTO algorithms can be found in Table 2.

##### ZigBee

The ZigBee specification defines a static RTO value for end-to-end transmissions *RTO_ZB_*, which is calculated by:

(2) $$RTO_{ZB}=0.05\;s\times (2\times \text{nwkcMaxDepth})+0.1\;s,$$

where the *nwkcMaxDepth* parameter denotes the maximum depth of the network. As a result, the RTO values used by ZigBee are constant and the same for all of the nodes of a network. If a retransmission is necessary, no RTO backoff mechanism, such as a Binary Exponential Backoff (BEB), is applied. Hence, the same RTO value is used for both the initial and the follow-up RTOs. The maximum depth of the experimental scenario described in Section 5 was measured and found to be 14. This leads to an RTO value of 1.5 s for the ZigBee protocol stack investigated in the evaluated testbed according to Equation (3).

##### ZigBee + Backoff

Since the use of backoff is common in RTO algorithms and to be able to analyze how adding a backoff affects the performance, the tests are also carried out with an alternative RTO algorithm that adds the BEB mechanism to the default ZigBee RTO algorithm. This algorithm will be referred to as “ZigBee + Backoff”, and the RTO value calculated by this algorithm is referred to as *RTO_ZB-BO_* in the evaluations.

##### CoAP

CoAP is a Representational State Transfer style (RESTful) protocol that allows the manipulation of resources between clients and servers, such as sensor measurements or actuator states, and is developed for constrained devices [36]. Amongst others, it is designed to be used in WHANs [37]. As in ZigBee, end-to-end ACKs are used to implement reliability at the APL layer. Therefore, the approach of CoAP to calculate an RTO value is evaluated in this paper as an alternative to the ZigBee approach. In CoAP, the initial RTO value for a transmission, *RTO_CoAP_*, is randomly chosen from an interval, where:

(3) $$RTO_{\text{CoAP}}=[2\;s,3\;s].$$

This relatively large initial value has been chosen to cope with high delays that may be observed when communicating with devices over the Internet. Additionally, the action that is triggered by the data packet (for example, the request of a complex calculation) may have a large processing delay. To avoid further network congestion, the follow-up RTO values are doubled with each retransmission in CoAP.

##### Cross Layer (CL)

In this more dynamic approach, we propose to use the NWK layer information to calculate the RTO value for a transmission. The approach consists in measuring the round-trip time (RTT) during the route establishment procedure (*RTT_RE_*) and using it to calculate an RTO value, *RTO_CL_*, by multiplying the RTT measured with a constant *K*, which is set to four by default. Hence,

(4) $${\text{RTO}}_{CL}=4\times {\text{RTT}}_{RE}.$$

*RTT_RE_* measured during the route finding procedure is an approximation of the RTT expected for the subsequent data transmissions. To avoid possible congestion of the network, the BEB method is applied. This type of RTO mechanism cannot be used by RFDs for data transmission, since they do not establish routes. Thus, the CL RTO mechanism is only applied to data transmissions of FFDs, while the RFDs use the default ZigBee RTO mechanism for this approach.

##### RFC 6298

TCP uses a more complicated algorithm to calculate the RTO value, as defined in RFC 6298 [38]. A smoothed RTT (SRTT) and a RTT Variance (RTTVAR) are maintained and used to calculate the RTO value, *RTO_TCP_*. The RTT measurements are taken according to Karn's algorithm [39]. The initial RTO value, according to the RFC, is 1 s by default. In our evaluations, it is set to 1.5 s to be identical to the value ZigBee uses. Additionally, a minimum RTO value is strongly recommended by the RFC. This led us to decide to use such a minimum RTO with a value of 1 s. The BEB method is employed for retransmissions in this algorithm.

## 4. Reference Scenarios

This section gives a detailed explanation of the WHAN reference scenarios evaluated through testbed experiments. These scenarios have been derived from the ZigBee HA profile [14] and the HA routing requirements defined by the IETF ROLL working group [1]. In addition to the basic test scenario described in this section, alternative network scenarios are defined in Section 7 to evaluate our findings in different topologies and for different transmit power settings. The design of the scenarios has been split into three main aspects: the logical roles of the nodes, the network traffic patterns and the evaluation setup. In the following, these scenario aspects are explained in detail.

### 4.1. Roles of the Nodes

From a use-case point of view, the WHAN setup represents wireless nodes associated with one or several electronic devices of everyday use. The nodes thus are commonly mains-powered [1]. The devices connected to a node in a WHAN may be sensors, actuators, controllers and other kinds of appliances. The wireless nodes are possible sources and destinations of information transmitted in the network. We define three roles for the nodes that cover most of the typical WHAN use cases:

(Role A) Nodes that have only a one-to-one relationships with other nodes of the network, for example, a light switch and a light bulb.
(Role B) Nodes that have one-to-one relationships with certain nodes of the network and that also produce data for a destination of many-to-one traffic. An example is a node with a motion sensor that causes an alarm for a security center upon noticing movement, while the node also periodically sends the measurements of a temperature sensor to a heating, ventilation and air conditioning (HVAC) system.
(Role C) Nodes that have one-to-one relationships with certain nodes of the network and are the destinations of many-to-one traffic. An example is an HVAC controller that collects temperature information and that is able to remotely control heaters, ventilators, *etc*.

### 4.2. Traffic Patterns

In WHAN scenarios, the amount of user data transmitted is usually small and, most of the time, only consist of a few bytes [1,14]. The actual amount of traffic depends on the number and the type of WHAN devices and how frequently they are used. To evaluate the performances of the network settings investigated, the degrees of device activities are varied in the network, as well as the roles of the devices. As a result, three different traffic scales are defined for the evaluations: low, medium and high traffic. This allows one to observe how the WHAN performs under a variety of situations and reveals how the performance of a certain communication protocol stack configuration scales with increasing traffic load. Two possible causes for the generation of data are considered:

(1) Aperiodic events: An aperiodic event can be, for example, an alarm generated by a motion sensor or by the command to turn on/off a device caused by toggling a switch. The amount of bytes that it takes to encode the information that needs to be transmitted is assumed to be three bytes. The total amount of bytes for the payload and the headers of such a message fits in the payload of an IEEE 802.15.4 packet, only requiring transmission of a single frame.
(2) Periodic events: For example, a periodic event can be a heartbeat signal from the nodes of a network that needs to be sent to a security center regularly or temperature information that is periodically collected and reported to an HVAC controller. The messages include aggregated information, such as sensor data and/or plain text, as part of status reports or log files, to name some examples. As a consequence, the total amount of bytes of a periodical event is assumed to surpass the maximum payload available in one MAC frame. Thus, three packets are assumed to be generated for a periodic event.

Depending on the roles of the nodes described in Section 4.1, the type of traffic they generate is defined as shown in Table 3. For example, a light switch (Role A) may only generate aperiodic events, whereas a temperature sensor (Role B) would generate periodic events for temperature statistics, but it also would generate an aperiodic event if the temperature exceeds a certain threshold. Aperiodic events always generate data for a random destination within the network.

Periodic events are generated with a fixed interarrival time of 60 s that does not vary with the traffic scale. Instead, the number of nodes producing periodic traffic is varied to achieve different traffic loads in the network. The interarrival time values for aperiodic events, *t_s_*, are defined to follow an exponential probability distribution function, the individual values of which can be generated through the use of a uniformly random variable, R, with the formula:

(5) $$t_{s}=\frac{-\text{ln}(1-R)}{\lambda },$$

where *R* is uniformly distributed between zero and one and λ is the average number of events per minute. Different traffic scales are achieved by varying λ and the number of nodes generating the aperiodic event data. For low, medium and high traffic loads, λ is defined to be 0.75, 1.25, and 1.75, respectively.

### 4.3. Evaluation Setup

The evaluations are carried out in a real testbed composed of 57 TelosB motes [40], for which the employed operating system is TinyOS [41]. We implement the fundamental mechanisms and parameters of ZigBee's communication protocol layers to carry out the evaluations. In contrast to commercial implementations of the ZigBee protocol stack, like BeeStack, with a fix set of changeable options and settings, our stack implementation allows us to freely modify all of its parameters and mechanisms. There may exist differences between the performance of our implementation and that of commercial ZigBee implementations. The TinyOS implementation of the protocol stack introduced in Section 3.2 can be accessed at [42]. The nodes are attached to a wooden grid of 8.1 m by 4.5 m that hangs *ca.* 0.5 m from the ceiling. All nodes are connected to a USB-tree that allows communication with a desktop PC to coordinate the experiments and to gather data during the evaluations. The grid is located in a laboratory, which contains objects of different heights and sizes that can be found in a house or an office environment. Physical phenomena in such environments, like multipath propagation and signal scattering, are effects observable in a WHAN environment that are difficult to reproduce realistically in simulators. Furthermore, it is necessary to take into account that the physical channel conditions of a WHAN may change over time. These changes affect the behavior of the network and happen due to several reasons, such as the interference caused by other devices operating at the same frequency band as the one used in the WHAN. Further reasons for varying physical conditions include moving objects or persons. The testbed chosen for experimental evaluations exhibits these requirements.

To imitate the typical characteristics of WHANs, the sensor grid needs to be configured adequately. The radio offers 32 transmit power levels that range from zero (lowest) to 31 (highest). In order to assure that connections with multiple hops are part of the evaluations, we configured the transmit power level to 1, which corresponds to a transmission power of –33.0 dBm. In the alternative scenarios defined in Section 7, we carry out evaluations also using a transmit power level of 2. Furthermore, the nodes are configured to work on Channel 26 of IEEE 802.15.4, avoiding most of the possible Wi-Fi interference.

**Figure 3.**
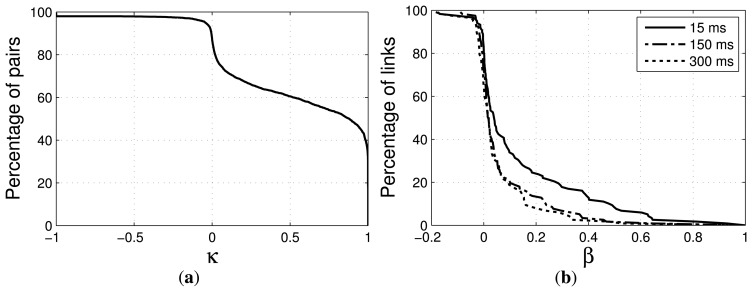
(**a**) ccdf of *κ* for link pairs in the testbed for a transmission power of about –25 dBm; (**b**) The corresponding ccdf of *β* for different inter-packet arrival times.

The real radiation patterns of the omnidirectional antennas vary from node to node, causing significant differences in transmission and interference ranges. Because of that and due to the spatio-temporal effects of the environment, even if all nodes are distributed equidistantly in a symmetric pattern, the links and link qualities between nodes vary significantly, offering a broad repertoire of characteristics that are typical for WHANs. The average number of hops between two randomly chosen nodes with this configuration lies below four, with a transmit power of 1, and below two, for a transmit power of 2, which captures the most common cases in WHAN scenarios [2]. The wireless links of the testbed can be characterized by the *κ*- and *β*-factors, which indicate the inter-link reception correlation and the link burstiness, respectively [43,44]. The *κ*-factor indicates to what degree the receptions of a packet on different links are correlated. The *β*-factor indicates to what extent a link of intermediate quality (10%–90% LDR) switches between poor and good delivery. The complimentary cumulative density function (ccdf) of the *κ*-factor of the testbed is depicted in Figure 3a. Nearly 50% of the links pairs in the network have a *κ* > 0.8. This characteristic is similar to the one of other testbeds analyzed in [43]. On the other hand, we measured low *β* values on the majority of the links, as can be seen in Figure 3b. This indicates that packet deliveries on the links of the network are mostly independent. The low degree of burstiness can be explained by the fact that measurements are carried out during nighttime, where the interference level or other causes of burstiness are low.

The measurements are carried out for different scenarios by varying the number of nodes and changing traffic patterns. In this work, we define three different topologies: the basic topology, and two alternative topologies, namely the dumbbell and the square topologies. In the basic topology, 52 nodes are configured to be FFDs and five nodes to be RFDs with the previously introduced configurations of the transmission channel and transmit power level. From the results obtained in the basic topology (see Section 5), two recommended protocol stack configurations are derived. In Section 6, we show that these settings improve network performance considerably. Eventually, these recommended settings are evaluated in Section 7 with different scenarios using the dumbbell and the square topologies and also with an increased transmission power setting of the nodes in all topologies. The details of the alternative scenarios can be found in Section 7.

**Figure 4.**
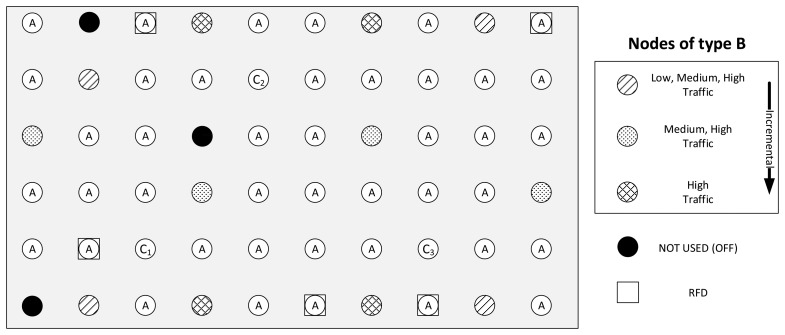
The testbed layout and Roles A, B and C assigned to the nodes of the testbed for the basic topology. Nodes that are not of Role B or of Role C at a certain traffic scale are of Role A.

The basic topology is illustrated in Figure 4. In the low traffic scale, four nodes of type B generate periodic traffic for the destination node C_1_, whereas all other nodes are of type A. The amount of nodes with role B increases linearly as the traffic scale increases (eight nodes at medium and twelve at high traffic scales). For each added group of B nodes, a new destination for their periodic traffic is added: C_2_ for medium traffic and C_3_ for high traffic. The additional B and C nodes are included in the grid by replacing the corresponding nodes with an A role. The five RFDs represent battery powered devices that are used sporadically, such as light switches. They generate aperiodic traffic with the arrival rate defined for the low traffic scale in all analyzed scenarios and are not the destinations of packets from other nodes, except for end-to-end ACKs. The RFDs are configured to sleep until an event that requires a data transmission causes them to wake up. After transmitting the data, and while waiting for an end-to-end ACK, RFDs will poll their associated FFDs at a rate of 500 ms per poll. After each poll, RFDs enter the sleep state until the next scheduled poll. If an RTO at the APL layer is triggered, the device wakes up and polls the associated FFD for the end-to-end ACK for which the RFD is awaiting. If no end-to-end ACK is available, the RFD retransmits the packet and continues with the cycle of sleep and poll. After receiving the ACK, the transmission is considered successful, and the RFD will sleep until the next data transmission. In addition, and in compliance with the ZigBee HA profile, RFDs will wake up at least once every minute to poll their associated FFD for data. The black node in the lower, left corner is used to align the internal timers of the nodes by sending synchronization messages at the beginning of a test run. Two other nodes (*i.e*., the remaining black nodes in Figure 4) were chosen randomly to be left unused with the goal of adding further irregularity to the grid, at the same time leading to areas of low connectivity.

Experiments in the grid are done during nighttime, where very low human activity and external wireless interference is observed. A relatively steady physical environment facilitates the comparison of different system parameter sets evaluated over time. Still, there may be small variances in the channel conditions when doing the experiments at different times. For this reason, to test a single configuration of the communication protocol stack, in total, 43 test runs are carried out, which are distributed over several days and add up to a total test duration of eight hours for each configuration.

At the beginning of each test run, all nodes are reset and the internal timers for aperiodic or periodic events are initialized. After completing a test run, the seeds of the nodes' random number generators are altered. Varying the seed changes the behavior of the timers for aperiodic events and the destinations selected for aperiodic data transmissions. The following section presents the results of the evaluation carried out in the basic topology.

## 5. Evaluation Results: Influence of Each Layer to the Overall Performance

This section presents the results of the evaluations of the ZigBee stack configurations presented in Section 3.2, which have been carried out within the basic topology scenarios described in Section 4.3. At the MAC layer, the impact of the maximum number of MAC layer retries is evaluated. The effects on the performance of using different routing metrics at the NWK layer are also analyzed in this section. Finally, the influence of the APL layer RTO algorithm is studied. Each set of measurements for a specific layer is carried out, while the rest of the communication protocol stack is configured with the default settings (see Section 3.2).

The effect of changing the parameters and mechanisms of the communication protocol stack, as well as introducing new mechanisms is measured in terms of three main performance metrics: the end-to-end PDR, the end-to-end delay and energy efficiency. While the goodput is mainly considered to be an interesting performance indicator for the transmission of large files, it may not have the necessary expressiveness in a WHAN, where the traffic is sporadic and of relatively low volume. The PDR is calculated by counting the total number of successfully delivered packets at the APL layer and dividing it by the number of packets created by the source APL layer entities. The PDR is an important indicator, since it describes how successful the communication protocol stack is in transferring packets through the network. On the other hand, the end-to-end delay is the total time between the transmission of an APL layer packet and its reception at the destination node. The end-to-end delay indicates the time it takes for the network to react to events, such as the push of a button or the triggering of a motion sensor. To measure the end-to-end delay, we use timestamps, taking advantage of the fact that all nodes in the network are synchronized (see Section 4.3). The energy efficiency indicates the average energy that is consumed by the RFDs to deliver one bit of useful application payload to corresponding destinations. The energy consumption is calculated by observing the behavior of the end devices, in particular by measuring the durations of different states and applying the energy consumption values listed in [45] for each state. Since we employ a different transmit power level, we measured the energy consumption of our nodes at the transmit state (*I_tx_*).

### 5.1. MAC Layer

**Figure 5.**
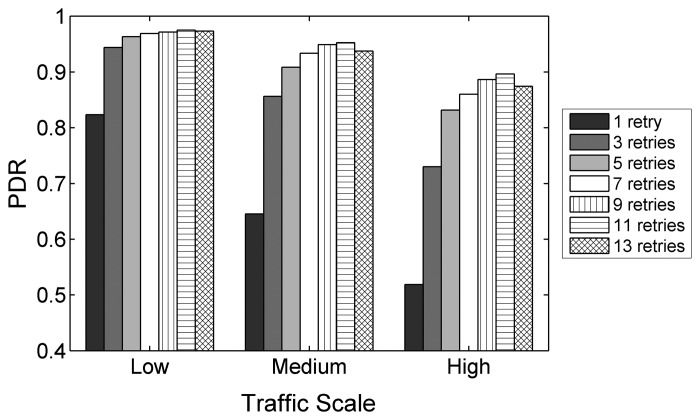
The overall packet delivery ratio (PDR) for different values of *macMaxFrameRetries* in different traffic scenarios.

**Figure 6.**
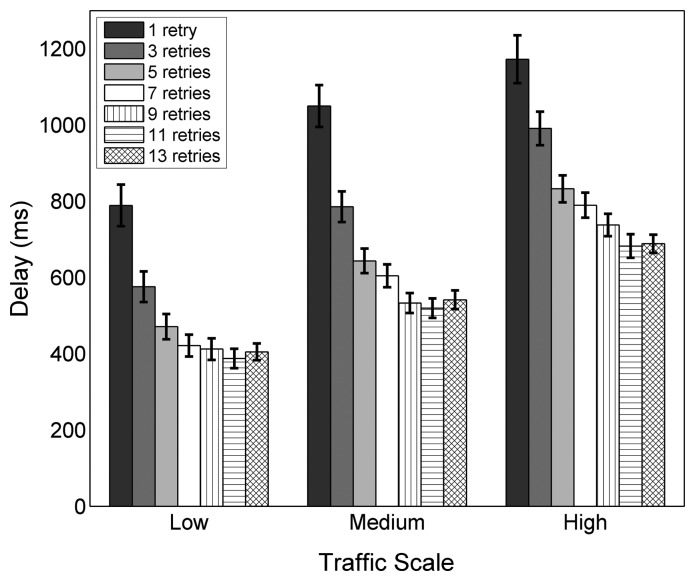
The average end-to-end delays observed with the corresponding 95% confidence intervals for different values of *macMaxFrameRetries* in different traffic scenarios.

**Figure 7.**
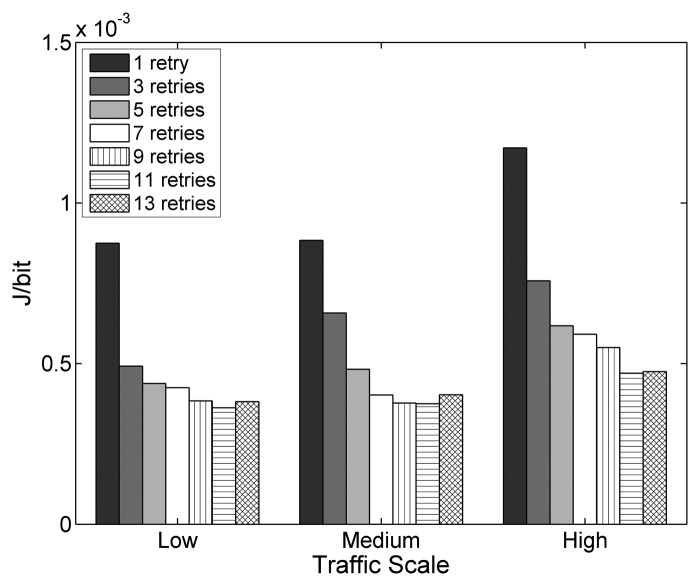
The average energy efficiency for different values of *macMaxFrameRetries* in different traffic scenarios.

The experiments with different values of *macMaxFrameRetries*, the parameter that defines the maximum number of MAC layer retransmissions, shows an important correlation between the setting of this parameter and the network performance. Changing *macMaxFrameRetries* affects the overall PDR noticeably, as depicted in Figure 5. As seen in the figure, for the values in the allowed value range from zero to seven, as specified by IEEE 802.15.4 and hence by ZigBee, a higher amount of allowed retransmissions results in a higher PDR. With the default configuration of three, the overall PDR decreases from 94.3% to 85.6% and to 72.9% for low, medium and high traffic, respectively. Moreover, the difference in overall PDR between the default configuration that allows three retransmissions and the two alternatives of using one and seven retransmissions, respectively, becomes larger with increasing traffic. It reaches its peak value at high traffic with a relative degradation of 28.9% when only using one retry and a significant relative improvement of 17.8% when using seven retries. The average end-to-end delay and the energy efficiency evolve correspondingly, as shown in Figures 6 and 7. While the delay is relatively high for a low number of allowed retransmissions, it decreases as more retransmissions are allowed. The energy efficiency improves similarly, as the energy consumption per bit decreases with an increasing value of *macMaxFrameRetries*.

**Figure 8.**
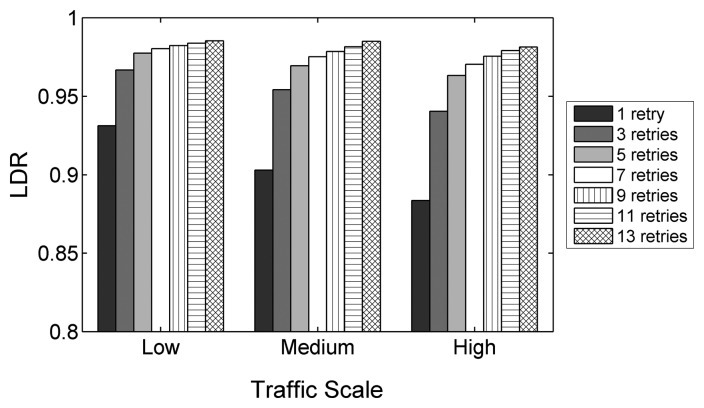
Link delivery ratio (LDR) for different values of *macMaxFrameRetries* in different traffic scenarios.

The reason for these results can be derived from the average data link delivery ratio (LDR), which depends on *macMaxFrameRetries*. Figure 8 shows that the LDR for different traffic scales increases rapidly with values above one for *macMaxFrameRetries*, flattening out with higher values when considering the allowed range of values for this parameter. As the traffic increases, the benefit of augmenting the number of allowed retries is higher, as packet losses due to intranet work interferences and collisions become more frequent.

Since it is necessary to transmit packets over multiple links, the LDR has a major impact on the overall PDR, determining with which probability the packets reach the destination node. The end-to-end delay similarly depends on the LDR. In a theoretical case of a 100% LDR, the end-to-end delay only depends on the number of hops between the source and the destination and on the time spent at each hop. In a practical case, links are lossy and the LDR adopts lower values, like the ones observed during the experiments. If a loss happens, which is more probable in links with lower LDRs, the APL layer end-to-end reliability mechanism will retransmit the packet. However, this requires the source node to wait for the RTO timer of the APL layer to expire, adding significant delay to the transmission. Therefore, as the LDR decreases, the end-to-end delay increases. The energy efficiency shows a similar improvement as PDR and delay for an increasing amount of allowed retransmissions. RFDs apply duty cycling to reduce the current consumption, which means that they spend an important amount of time in the sleep state, where very low energy is consumed. In any other state, the RFDs consume more energy, especially when transmitting and receiving data. The sleep state is interrupted for data packet transmissions (including retransmissions) or to poll the associated FFDs for end-to-end ACKs by sending a data request and waiting for a reply. The amount of data transmissions and polls therefore has an important impact on the energy consumption. Packet losses that lead to APL layer retransmissions and that cause the RFDs to waste polls while waiting for end-to-end ACKs that never will arrive decrease the energy efficiency.

**Figure 9.**
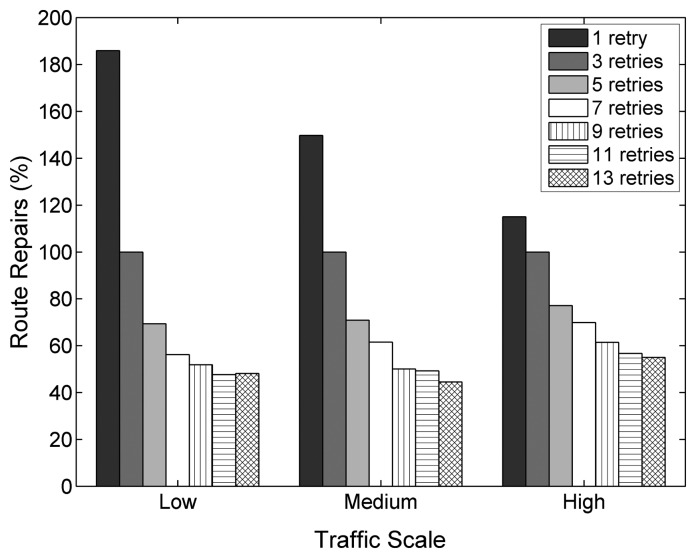
Relative amount of route repairs for different values of *macMaxFrameRetries* taking the default value of three as the base.

On the other hand, an important cross-layer mechanism depends on the value of *macMaxFrameRetries*. According to the ZigBee specification, an unsuccessful MAC layer transmission (after using all retries) is interpreted as a link failure at the NWK layer, which triggers the route repair mechanism. As a consequence, the network is flooded with RREQs, adding a significant amount of traffic to the network that may decrease the performance of ongoing transmissions. A high amount of allowed retries reduces the overall amount of route repairs that are initiated during the tests (see Figure 9), decreasing the control message overhead and reducing the network congestion.

When using values higher than those allowed by the specification (9, 11 and 13 retries), at all traffic scales, further improvements are observable for nine and 11 retries. However, with a maximum of 13 retries, the PDR and energy efficiency drop and the delay increases. With 13 allowed retries, the congestion created by repeatedly trying to transmit frames at medium and high traffic rates becomes significant, leading to a degradation of the overall performance, as one-hop delays become larger and buffer overflows occur more frequently due to increased buffer occupation times. This also reflects in the energy efficiency, which increases for nine and 11 retries and decreases for 13 retries.

Judging from the results obtained in the experiments, it is highly recommended to increase the default value of *macMaxFrameRetries* for ZigBee HA profile systems to the maximum the specification allows, as it benefits the PDR, delay and the energy efficiency for end-to-end transmissions. Moreover, the allowed value range of *macMaxFrameRetries* should be increased, as further performance gains can be achieved by using values from the extended value range.

### 5.2. NWK Layer

This subsection studies how the overall network performance of the commonly used BeeStack routing metric compares to the performance of the HC, PLF and Path-DR routing metrics. The routing metric only is relevant for the FFDs that are participating in the routing. RFDs are not capable of routing; they are associated over a one-hop relationship to an FFD that is responsible for routing messages to and from the RFD. However, the routing metric also has a relevant impact on the performance of the end devices, as will be shown later, since all of the messages they are sending and receiving are routed by FFDs through the network.

**Figure 10.**
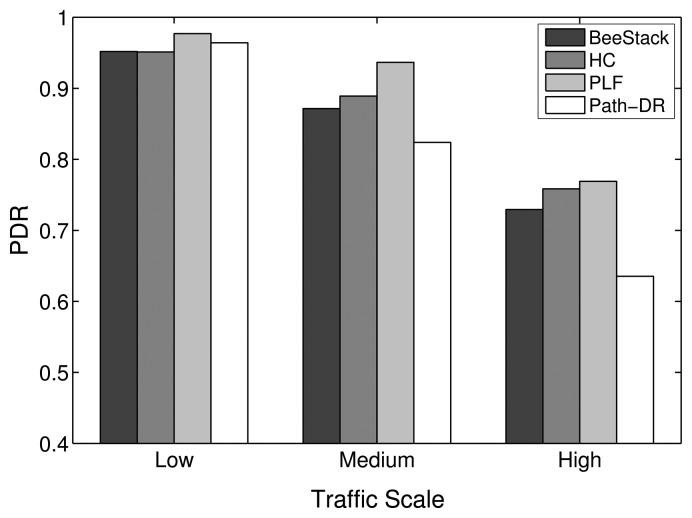
A comparison of the overall PDR for different routing metrics and traffic conditions.

**Figure 11.**
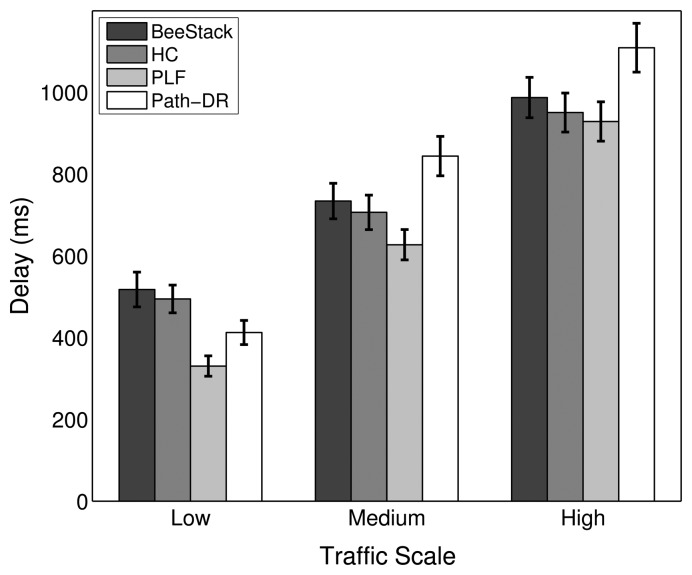
A comparison of the average end-to-end delays with 95% confidence intervals for different routing metrics and traffic conditions.

**Figure 12.**
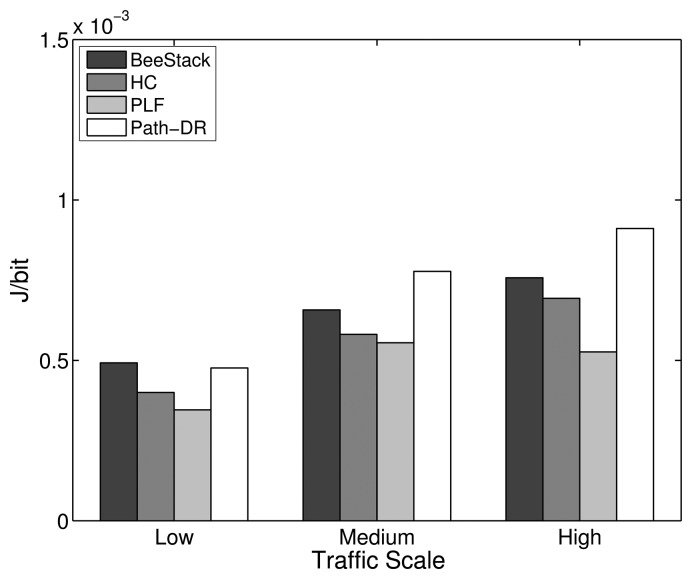
A comparison of the average energy efficiency for different routing metrics and traffic conditions.

Figure 10 shows that the PLF metric always performs best in terms of overall PDR. The HC, BeeStack and Path-DR metrics follow in the given order, except for the low traffic case, where the Path-DR metric performs the second best. A strong degradation of the Path-DR performance can be observed for higher traffic loads. A higher PDR is correlated with a lower end-to-end delay, as can be seen in Figure 11. A similar dependency has been observed in the previous subsection between the PDR for the maximum MAC layer retransmission parameter and the resulting end-to-end delay. As explained in the analysis of the MAC layer results, the energy per bit should decrease with shorter end-to-end delay and higher PDR. The measurements confirm this assumption, as can be seen in Figure 12.

Detailed investigations have been carried out to find out the reasons for the performance differences between the considered metrics and for the unexpected worse performance of Path-DR for the medium and high traffic scales. The main reason for the differences in the performance of the metrics lies in the characteristics of the chosen routes. The decision criteria of the routing metrics determine the number and the quality of the links of the resulting paths.

**Figure 13.**
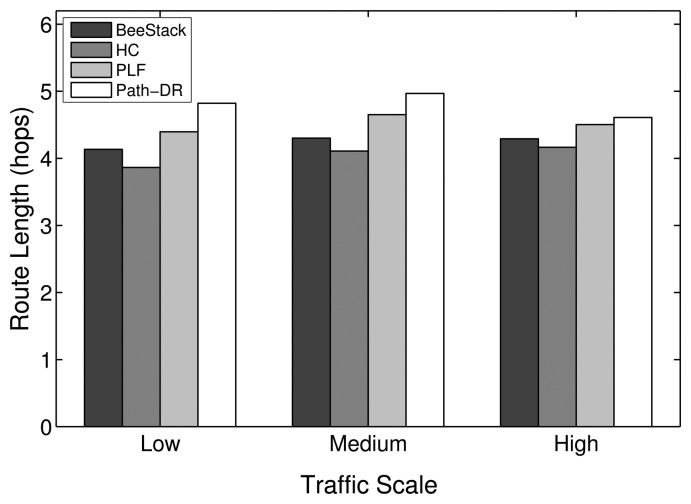
Average lengths of routes chosen by different routing metrics and traffic conditions.

In the experiments, the HC metric results in the minimum average path length, as expected and as seen in Figure 13, while the average length of routes chosen by Path-DR is the largest one. The average lengths of the routes chosen by the BeeStack and the PLF metrics lie between the ones chosen by the HC and Path-DR metrics. This can be explained by the fact that the BeeStack and PLF metrics apply an additive formula to calculate the path costs. Contrariwise, the Path-DR metric applies a multiplicative formula to calculate the expected PDR. Due to the multiplicative path cost calculation, Path-DR prefers longer routes with higher quality links over shorter routes with lower quality links. As an example, a route with a multitude of hops that have an expected LDR of almost 100% is preferred by the Path-DR metric over a single hop with a low expected LDR. This is unlikely for the BeeStack and PLF metrics, since each hop increments the path cost, making longer routes expensive. This behavior causes the routes chosen by the PLF and BeeStack metrics to be shorter than those selected by the Path-DR metric.

The end-to-end PDR of a route strongly depends on the link qualities along the route. Except for the HC metric, the routing metrics base their choice of routes on the measured link qualities. As indicators of the link qualities, LQI values are used, since a high LQI normally is correlated with a high LDR. Therefore, routing metrics that base their decision on measured link qualities expect a high end-to-end delivery ratio over these routes. The performance of the routing metrics depends on the mapping from LQI to link costs or expected LDR values. The BeeStack and PLF metrics use a coarse granularity mapping that converts LQI values into link costs, being three steps in the BeeStack metric and seven steps in the PLF metric. On the other hand, the Path-DR metric defines 61 steps when transforming the LQI values into expected LDR values. Metrics that use finer granularity mappings are able to detect a wider spectrum of link qualities and identify high quality links with higher certainty.

However, the route length and the link qualities of a route alone do not decide how well a transmission over this route performs. An important factor that is not considered by any of the metrics is found to be network congestion. A higher amount of traffic increases the degree of contention among nodes inside the network, leading to interference and collisions. This directly affects the average number of retries used by the IEEE 802.15.4 MAC layer when transmitting unicast data frames. Table 4 demonstrates that the number of tries observed for each metric increases with the traffic, which is an indicator of congestion. Experiments have shown that LQI values measured with and without internal network interference are almost the same. One reason could be that only the link qualities from the correctly received packets are considered by the link quality-aware metrics, while the packets with the errors that result from collisions or interference are not considered. This leads to the conclusion that the measured LQI values do not serve as indicators for network congestion. Because of this phenomenon, the link quality-aware metrics are not able to identify congested links. If a metric tends to choose longer routes, like the Path-DR metric does, the fact that additional transmissions are necessary with each hop can lead to further congestion and to a lower end-to-end performance.

**Figure 14.**
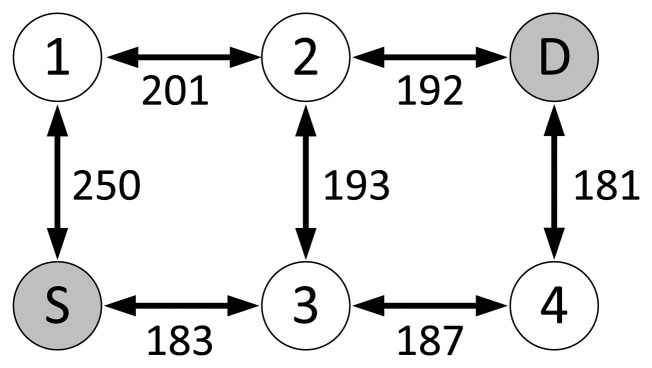
Example topology, where the Path-DR metric only finds one best route between the source (S) and destination (D) (S-1-2-D), while the ZigBee path cost metrics (HC, BeeStack and PLF) find three routes of equal cost (S-1-2-D, S-3-2-D, S-3-4-D). The numbers on the links represent the LQI values of the links. Note that all ZigBee path cost metrics will assign the same cost to all of the links shown in this example (see Figure 2).

Furthermore, the experiments have shown that the link quality-aware metrics will more likely overuse the links with high LQI values, leading to local congestion. This issue especially affects the Path-DR metric. Figure 14 depicts an example where the Path-DR metric only finds one best route, while the evaluated ZigBee metrics are able to find three best routes of equal costs over different links. The restriction of Path-DR to routes of very high quality, due to its fine link and route cost granularity, increases the amount of transmission attempts over them and is an important source of congestion. As a consequence, the Path-DR metric performs poorly at medium and high traffic scales, when compared with the ZigBee metrics (including the HC metric), as shown in Figures 10, 11 and 12. In contrast, the link and path cost granularity of the BeeStack and the PLF metrics is significantly coarser than that of Path-DR. This increases the probability of determining the same path cost for routes that have slightly different link qualities when the BeeStack and the PLF metrics are used. Therefore, the BeeStack and PLF metrics provide greater path diversity, which naturally tends to reduce network congestion.

The PLF metric chooses links with high LQI values, but in contrast to the Path-DR metric, it does not restrict its set of available routes to a small subset of optimal routes, as illustrated in Figure 14. The combination of the greater path diversity and sufficient link quality-awareness leads to a better performance of the PLF metric for all traffic scales. On the other hand, the BeeStack metric performs worse than the HC and PLF metrics. The coarse link and path cost granularity is one reason. Figure 2 shows that 70% of all LQI values are mapped to the same link cost, which means that the BeeStack metric assumes that all of the links in this large interval are of similar, if not equal, qualities. The second reason for the inferior performance may originate from the fact that the LQI value is obtained from RSSI measurements of the radio transceiver in the BeeStack implementation, instead of relying on correlation values. Finally, note that the HC metric is not link quality-aware and, thus, may select both high and low quality links, but it guarantees the shortest route.

**Figure 15.**
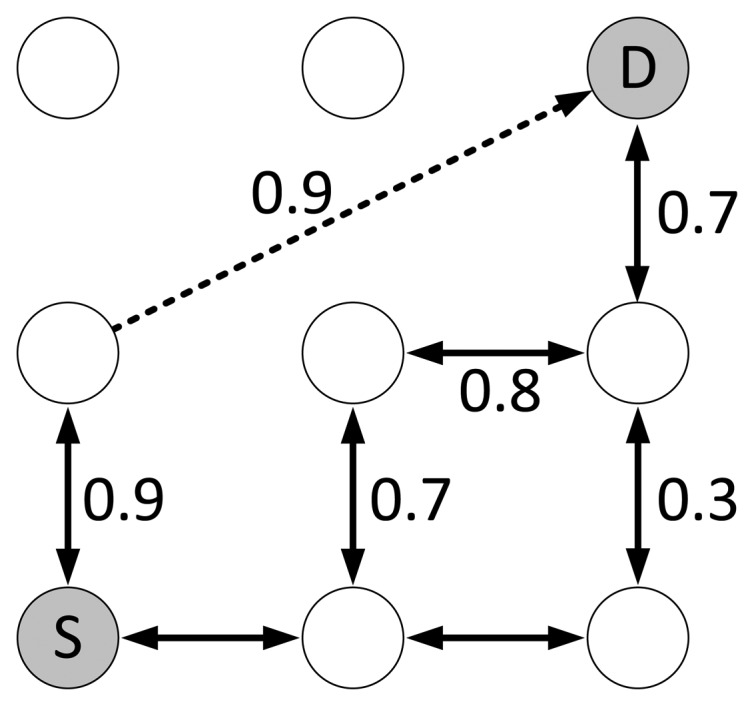
A network topology, where an asymmetric, unidirectional link of high quality (dashed line) is likely to be chosen by the routing metrics, even though its asymmetry impedes RREPs sent by the destination (D) from being returned to the source node (S). The numbers represent the LDRs of the links.

Another important issue observed throughout experiments in the testbed environment is the negative effect of asymmetric links on network performance. Similar observations have been made in the literature [46]. Normally, the forward and backward links of a route between two nodes are of similar qualities in terms of LDR or LQI. However, it has been observed in the testbed environment that approximately 6% of all links between nodes are strongly asymmetric. These links are normally of very high quality in one direction and have a very bad or no connection at all in the other direction. For metrics that take into account the link qualities of the forward route towards the destination, asymmetric links can result in unsuccessful route discoveries, as RREPs may not be able to return to the source node. Figure 15 illustrates this situation. In this scenario, a route discovery would not provide a valid route, even though there exists at least one. The path cost for a route that includes the asymmetric link would be better for all metrics than the path costs for the alternative routes that do not include asymmetric links. The issue of asymmetric links affects especially link quality-aware metrics. This leads to higher channel usage (all retries are spent to forward RREPs that never reach their destination nodes) and also to higher buffer occupation inside the nodes (the buffers are occupied longer, while retries are spent). While this phenomenon affects all of the analyzed link quality-aware metrics, the larger diversity of possible routes that are found by the BeeStack and PLF metrics augments the probability of finding a usable route with these two metrics. The Path-DR metric, however, has a lower probability of finding alternative routes, since the route diversity is much smaller, as illustrated in Figure 14. This is another reason for the Path-DR metric to perform badly for medium and high traffic scales. Because of these observations, we recommend using an LQI to link cost mapping for the ZigBee path cost metric of medium granularity, which allows one both to discard low quality links and to assure sufficient path diversity, like in the PLF metric.

There are several ways to address the problem of asymmetric links. One solution is to use asymmetric routing, where the forward and backward paths are not the same. This requires two separate route discoveries. The ZigBee Pro specification includes this feature as an optional mechanism. Outside the ZigBee specification, modifications of the default AODV, like R-AODV [47], implement asymmetric routing.

### 5.3. APL Layer

At the APL layer, we evaluate the impact on the end-to-end network performance of the five RTO algorithms that have been introduced in Section 3.2 in terms of overall end-to-end PDR and delay, as well as the energy efficiency of the RFDs. Using an adequate RTO value for APL layer data transmissions is important for the end-to-end performance, because of two main reasons. First, the probability of producing spurious retransmissions and, thus, contributing to network congestion increases if the RTO value is set too low. Second, the probability of detecting a loss incurring a large delay increases if the RTO value is set too high.

**Figure 16.**
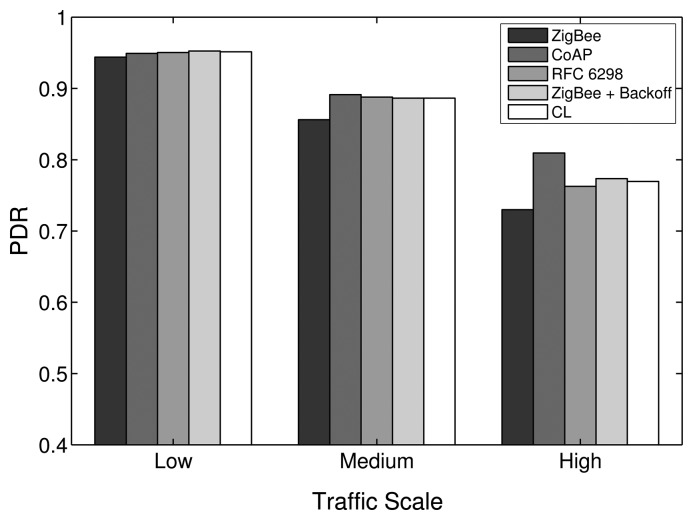
PDR results for different RTO algorithms.

**Figure 17.**
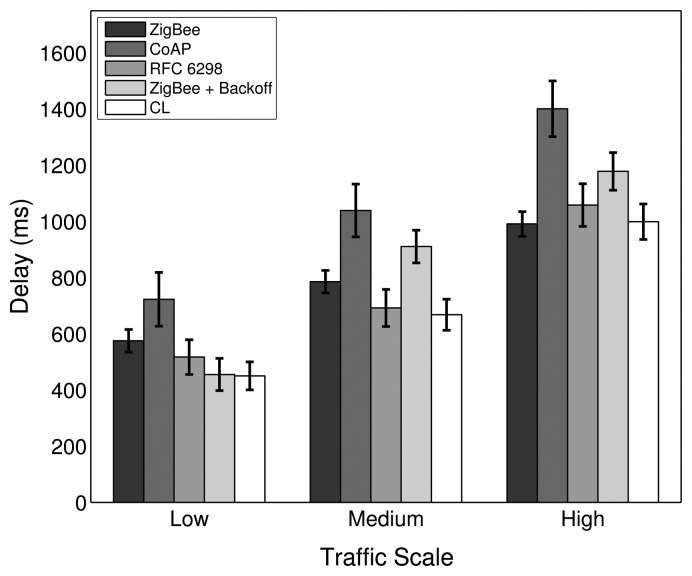
Average end-to-end delays with 95% confidence intervals for different RTO algorithms.

**Figure 18.**
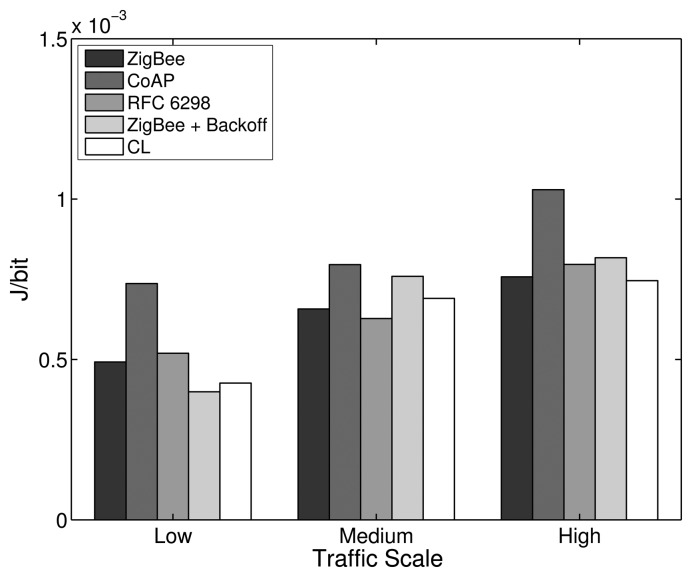
Energy efficiency for different RTO algorithms.

For the low traffic scale, all RTO algorithms show a similar performance, as the number of APL layer retransmissions needed is small. The similarities in the performances apply to the PDR, delay and energy efficiency metrics, as shown in Figures 16, 17 and 18, respectively. In the low traffic case, most of the end-to-end data transmissions are successful, and for most of them, the average RTT lies below the initial RTO values. As a result, the impact of the RTO algorithms on the end-to-end performance is rather small for the low traffic scale. The higher delay and lower energy efficiency observed when using the CoAP algorithm is an exception, as even a few packet losses cause the average delay and the energy consumption to increase noticeably due to the high initial RTO value used by this algorithm.

In the medium and high traffic scales, end-to-end delays get larger due to congestion, and accordingly, the average RTT is augmented. Together with a higher packet loss ratio, the frequency of RTO expirations increases noticeably. For example, when using the default stack configuration with the ZigBee RTO algorithm, compared to the low traffic case, the number of RTO expirations is augmented by 330% at medium traffic and by 813% at high traffic. As RTO expirations become more frequent, the impact of the RTO algorithms on the overall network performance becomes greater. The default algorithm is outperformed by all other algorithms in terms of PDR for medium and high traffic scales, as seen in Figure 16.

With the medium traffic scale, the average delay obtained with the default ZigBee RTO algorithm lies in between the delay values observed when using the other RTO algorithms. The CoAP algorithm delivers the best PDR performance, but suffers from the highest delays. Similarly, the ZigBee RTO algorithm with BEB has a larger delay than that of the default RTO algorithm. The PDR and delays of the TCP RTO algorithm and the CL RTO algorithm are very similar, performing better than the default algorithm. The energy efficiency of the different algorithms follows a pattern that resembles qualitatively the curve observed for the end-to-end delay.

For the high traffic scale, the CoAP RTO algorithm achieves the highest PDR at the cost of the highest average delay. The other alternative algorithms achieve a similar PDR, lying between the PDR of ZigBee and CoAP RTO algorithms. We observe that like the CoAP algorithm, the RTO expirations of the ZigBee RTO backoff grow exponentially and lead to a higher amount of polls when packets are lost and to a higher energy consumption. The RFC 6298 RTO algorithm performs slightly better, explainable by the lower average delay. The CL RTO algorithm has a low delay and a higher PDR than the default RTO algorithm. As a consequence, the energy efficiency of this algorithm is the highest one.

**Figure 19.**
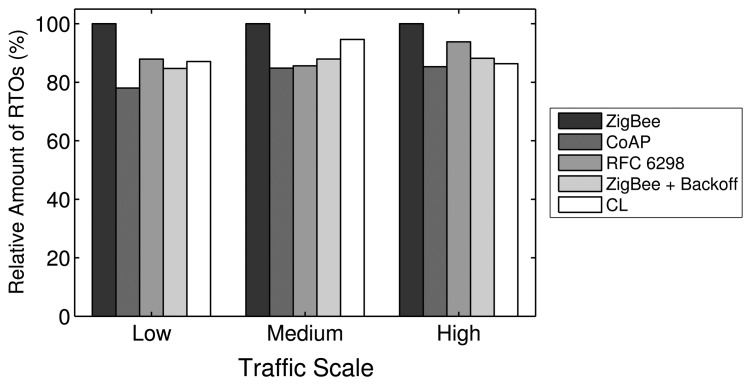
Relative amount of RTO expirations as seen from the application (APL) layer, when using different RTO value calculation algorithms, taking the default ZigBee RTO method as the reference.

When the default ZigBee RTO algorithm is used, under medium and high traffic load, the RTT frequently surpasses the 1.5 s of the initial RTO value. Since no RTO backoff is applied, two main effects are observable. If a packet really gets lost during a (re)transmission, the reaction time to the loss is constant, even after subsequent losses, contrary to the algorithms that apply a backoff. This assures a fast recovery from packet losses and helps to keep the delay low, even if the packets are lost consecutively. On the other hand, if the retransmission of the data packet is spurious and the network suffers from congestion, a quick retransmission may increase the congestion even further. This aggressive behavior comes at the cost of the lowest overall PDR and the highest amount of RTO expirations among the investigated RTO value calculation approaches (see Figure 19). By adding a backoff to the default algorithm, the ZigBee RTO algorithm with BEB achieves higher PDR at the cost of higher delays for medium and high traffic scales.

When compared to the default ZigBee RTO algorithm, the CoAP RTO algorithm delivers a noticeably better performance in terms of PDR. Because of the large initial RTO value, spurious retransmissions are not likely. This positively affects the amount of RTO expirations (Figure 19). The drawback of such a large initial RTO value is the large amount of time that has to pass until a packet loss is detected. Independent of the amount of traffic, this causes the average delay and current consumption of the CoAP algorithm to be the largest, as seen in Figures 17 and 18.

The performance of the CL algorithm heavily depends on the network condition during the route discovery and the RTT-multiplier *K*. For *K* = 4, the CL algorithm performs on average slightly better than the default algorithm in terms of PDR for all traffics and in terms of delay with low and medium traffic. The energy efficiency of the CL algorithm on average is very similar to the one obtained by the default algorithm. However, it is important to point out that the values calculated by the CL algorithm varies strongly with the route hop count. Since the RTO value is a multiple of the RTT in this case, short routes with a low end-to-end delay will have a relatively short RTO value compared to long routes with a large delay. These differences in RTO values cause the behavior of the CL algorithm to be adapted to the number of hops of a path. Overall, this algorithm provides higher PDR than the ZigBee algorithm, lower delay (except for high traffic), a similar energy efficiency and a lower amount of RTO expirations. Experiments with higher *K* values led to higher overall PDR, but also larger delay. Smaller values led to the opposite effect, offering lower overall PDR and a shorter delay. Choosing *K* to be four provided a good tradeoff in the considered scenarios.

The RFC 6298 algorithm performs slightly better than the default ZigBee algorithm at all traffic scales in terms of PDR, while the average delay is slightly smaller at low and medium traffic. At high traffic, the delay is larger for the RFC 6298 algorithm. In terms of energy efficiency, it performs slightly worse than the default algorithm in all traffic scenarios. In TCP, this algorithm is used to maintain an accurate estimation of the RTT during a transport layer connection. This normally involves the exchange of multiple packets between the endpoints of the transmission. Using this RTT information allows the sender to dynamically adapt the RTO value for subsequent transmissions to a specific destination. This means, however, that for end-to-end data transmissions with few packets, as are expected in a WHAN, the algorithm may not develop its full potential: the majority of events happen infrequently and only require a single data packet to be transmitted from the source to the destination node. On the other hand, when three packets are transmitted as a result of a periodical event (or even more packets, when adding up several events for one destination), the algorithm may actually do some RTT calculations to find an efficient RTO value. According to Karn's algorithm [39], RTT measurements may not be taken from retransmissions. While it is likely to get valid measurements at low traffic with a low amount of RTO expirations, the number of valid RTT measurements diminishes at medium and high traffic scales, where RTO expirations happen frequently. The ability to tune the RTO value to the RTT at low and medium traffic allows the RFC 6298 algorithm to achieve lower delay and higher PDR. At high traffic, the average delay changes from being lower to being higher than the one obtained by the default RTO method, due to a greater number of RTO expirations and the consequently applied BEB mechanism. To reduce the amount of RTO expirations and to increase the probability of getting valid RTT measurements at high traffic scales, the initial RTO value could be increased, as higher delays are expected. However, a higher initial RTO value (like the one used by CoAP) would increase the delay and reduce the energy efficiency if packet losses occur on routes that have a low delay. Overall, since the amount of valid SRTT calculations is low, the TCP algorithm mostly uses the default RTO value and the BEB; therefore, it cannot clearly outperform the other algorithms.

It can be shown that from the five RTO algorithms that have been analyzed, there is not an optimal one that provides the highest PDR, the lowest delay and the best energy efficiency at the same time. However, the CL algorithm offers a good tradeoff between these three performance parameters. The implementation of this algorithm is a simple alternative to the default ZigBee algorithm; hence, we consider the CL algorithm as an interesting possibility to augment the performance of the default ZigBee stack. Overall, the influence of the RTO algorithm on the overall performance is rather small compared to the impact achieved by modifying the number of MAC layer retries or the routing metric. However, the results show that the effect of the RTO algorithm increases with the amount of traffic.

## 6. Recommended Stack Configurations and Their Comparative Performance Evaluation

In a WHAN scenario, high PDR, as well as low delay and high energy efficiency are important. Hence, a protocol stack configuration used in this kind of environment should satisfy all of these requirements. The results from the previous section have shown that the default ZigBee stack configuration can be outperformed with alternative configurations in a WHAN for all traffic scales. Therefore, its default parameters and mechanisms need to be modified. In the literature, there exist proposals for mechanisms that adapt crucial protocol stack parameters dynamically to the current network state to improve network performance. An isolation layer [48] or pTunes [49] are examples for such proposals, while they propose approaches that disrupt the ZigBee architecture or are specific to tree topologies. However, in this study, we consider mesh topologies (see Section 3.2.2), and we evaluate alternative stack configurations that do not disrupt the ZigBee stack architecture.

The first, called Recommended-Compliant, is compliant with the ZigBee specification, *i.e.*, the parameter values lie in the allowed range and the protocol mechanisms are not changed. This configuration includes the following:

- The amount of allowed MAC layer retransmissions is set to seven, which is the maximum allowed by the IEEE 802.15.4 specification. Lower values have proven to deliver lower PDR, larger delay and worse energy efficiency, which is not desirable in a WHAN.
- The ZigBee path cost routing metric should be capable of choosing routes that perform well under all tested traffic conditions. According to the results presented in Section 5.2, the PLF metric fulfills these requirements.
- The default ZigBee RTO algorithm is used.

The second recommended stack configuration, called Recommended-Unrestricted, includes the use of parameter settings and mechanisms beyond the bounds of the ZigBee specification:

- The maximum amount of MAC layer retransmissions is set to 11, since the results showed that with this value, the performance increases even more than that of using seven retries. A higher value, however, led to a decrease in the performance.
- Again, the PLF metric is applied for the ZigBee path cost metric.
- The default ZigBee RTO calculation mechanism is replaced with the CL algorithm (*K* = 4), since it delivers a good performance with a good tradeoff between PDR, delay and energy efficiency for all traffic scales.

**Figure 20.**
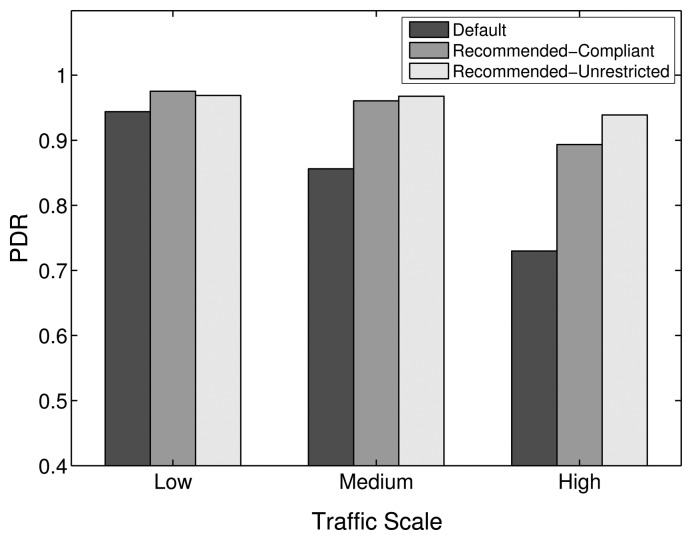
Comparison of overall PDR between default, Recommended-Compliant and Recommended-Unrestricted stack configurations.

**Figure 21.**
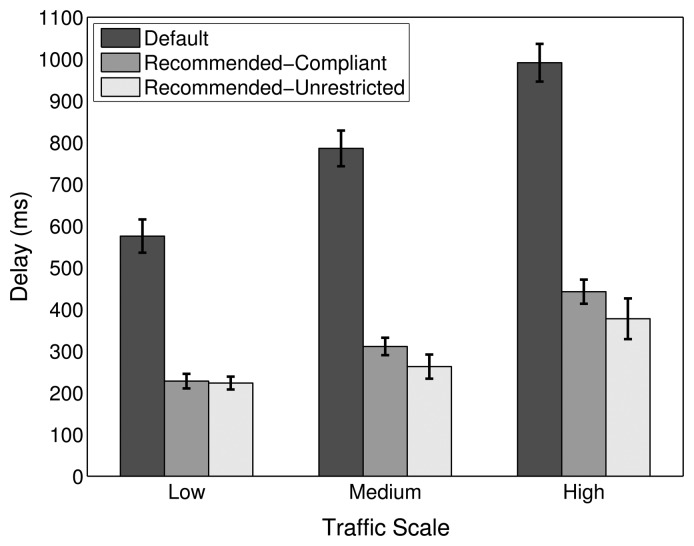
Comparison of average delays between default, Recommended-Compliant and Recommended-Unrestricted stack configurations, including 95% confidence intervals.

**Figure 22.**
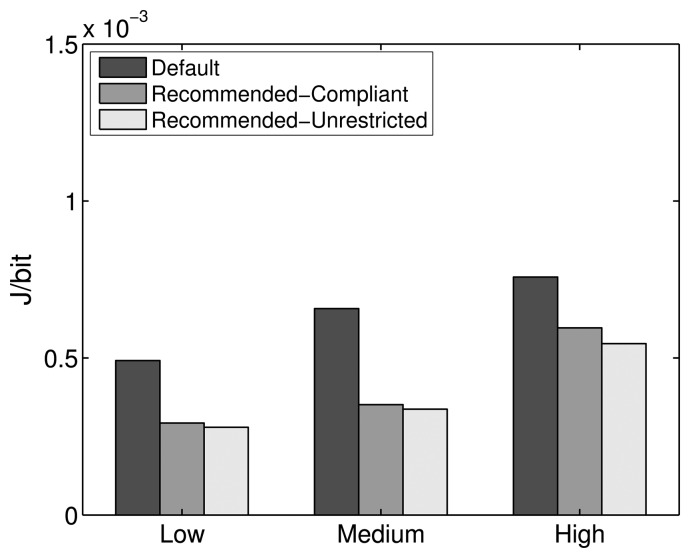
Comparison of average energy efficiency between default, Recommended-Compliant and Recommended-Unrestricted stack configurations.

Experiments have been carried out for the comparison of PDR, delay and energy efficiency performance of the default, Recommended-Compliant and Recommended-Unrestricted stack configurations, the results of which are shown in Figures 20, 21 and 22, respectively.

When compared to the default configuration of the ZigBee stack, the results with the alternative protocol stack configurations show a noticeable improvement in PDR, delay and energy efficiency. Increasing the amount of MAC layer retransmissions augments the one-hop reliability, which is especially important for higher traffic scales, where losses due to collisions and interference have to be compensated for. Furthermore, insisting in the transmission over a specific link, instead of considering it broken and doing a route repair quickly, is less costly in terms of PDR, delay and energy efficiency. Additionally, the PLF metric uses a LQI to link cost mapping with sufficient granularity, which provides a good path diversity at the same time. As shown in Figure 20, the PDR of the Recommended-Compliant configuration improves by 4.4%, 14.6% and 31.5% for low, medium and high traffic, respectively. At the same time, the delays decrease by 60.4%, 60.4% and 55.4%, as shown in Figure 21, and the energy efficiency improves by 40.5%, 46.5% and 21.4%. These results evidence significant network performance improvements, which show that the default ZigBee stack configuration for WHANs should be revisited.

With the Recommended-Unrestricted configuration, where the amount of allowed MAC layer retries is further increased to 11 and the default RTO algorithm is replaced by the CL algorithm, an additional improvement in the PDR performance is observable for medium and high traffic scales. In terms of PDR, the improvement achieved by the Recommended-Unrestricted configuration in comparison with the ZigBee default one is 4.1%, 15.3% and 33.6% for low, medium and high traffic, respectively. Correspondingly, the end-to-end delays decrease by 61.2%, 66.6% and 61.9%, while the energy efficiency improves by 43.2%, 48.7% and 28%. Hence, it has been shown that by relaxing the allowed range of settings for network parameters and by allowing the cross layer feedback from the routing layer to the application layer, a further PDR improvement of up to 2.1%, a delay improvement of up to 6.5% and an energy efficiency improvement of up to 6.6% are possible. The energy efficiency of the Recommended-Unrestricted settings stays very similar to the one of the Recommended-Compliant protocol stack configuration (Figure 22).

## 7. Performance Evaluation of the Recommended Stack Configurations in Alternative WHAN Scenarios

**Figure 23.**
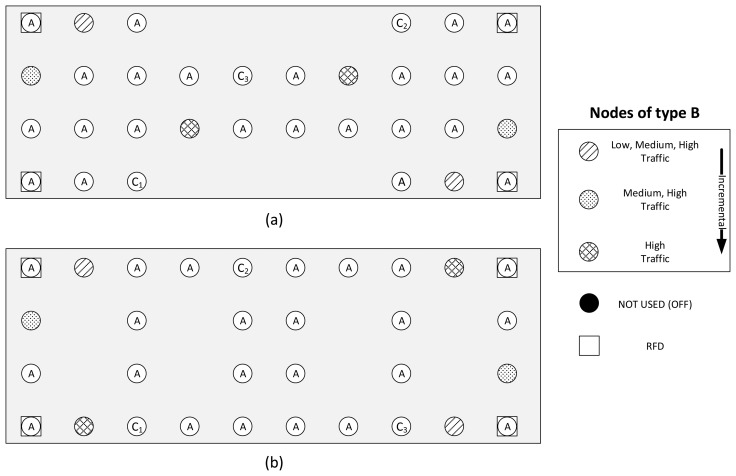
(**a**) Dumbbell and (**b**) square topologies, with roles as defined for the basic scenario.

In this section, we evaluate the performance of the default protocol stack configuration and the two recommended settings that have been introduced in the last section in alternative WHAN scenarios to cover a larger variety of possible network setups. First, we introduce two alternative network topologies that differ in size and layout from the basic test scenario and evaluate the performance of the default, Recommended-Compliant and Recommended-Unrestricted settings under these topologies. Second, we repeat these evaluations in all three introduced topologies with the transmit power level 2, corresponding to a transmission power of −28.7 dBm. For this transmission power, we measured a current consumption for the transmission state of *I_tx_* = 7.3 mA. We demonstrate that in all topologies, independent of the transmission power level, an improvement can be achieved with the two recommended protocol stack configurations. We introduce the dumbbell topology (where nodes in the central zone are likely to suffer a greater degree of congestion than in the basic topology), as well as the square topology, where there exists a smaller node degree and, thus, a lower amount of end-to-end connectivity paths, in comparison with the basic topology. Figure 23 shows the layout of the dumbbell and square topologies and the assignment of roles to nodes for different traffic scales. The roles are the same as the ones defined for the basic topology in Section 4.

We extracted the percentage improvement achieved by the recommended configurations over the default ZigBee stack configuration for different topologies and different traffic scales. For brevity, the obtained results are not presented in detail as in the evaluation section of the basic topology. The detailed results, including the figures for beta and kappa at a transmission power of −28.7 dBm, can be found in [42]. We present the improvement achieved for a given topology over high traffic scale in Tables 5 and 6 for a transmission power of −33.0 dBm and −28.7 dBm, respectively. Results confirm that the recommended settings are able to augment network performance without incurring any additional traffic overhead. As shown in Table 5, the overall PDR increases slightly in the alternative topologies, while the delay and energy efficiency improve noticeably. The lower performance improvement achieved by using the recommended configurations in the alternative topologies can be explained by the fact that in these scenarios, the network congestion is low, since the amount of nodes that generate traffic is almost halved compared to the basic topology. This results in a higher stability of the routes, less packets drops and lower one-hop delays. With the default protocol stack configuration, a high PDR is obtained. Still, the improvements provided by the recommended protocol stack settings are able to increase the overall PDR further. Less APL layer retransmissions and route repairs are necessary, causing a significant decrease in the average delay and an increase in the energy efficiency.

Table 6 shows the improvement over the default communication protocol stack settings of the Recommended-Compliant (Compl.) and Recommended-Unrestricted (Unrestr.) configurations for the increased transmission power setting. As for the lower transmission power, the recommended configurations again improve network performance. There is a noticeable improvement of delay and energy efficiency, while the PDR stays almost the same. We observe that by incrementing the transmission power, the connectivity of the network changes dramatically, as the radio transmission range increases noticeably and so does the number of neighbors of the nodes. As a consequence, the average amount of hops between two nodes is much lower. Further, we observe that the overall PDR is almost 100% for all topologies, independent of the amount of traffic. Therefore, the achievable performance improvements in terms of PDR are rather low. The improvement of delay and energy efficiency are higher, since the recommended protocol stack configurations reduce the amount of packet losses and, thus, the APL layer RTO expirations, which has a noticeable positive effect on the delay and the energy efficiency.

Overall, it has been shown that the recommended configurations for WHANs that have been derived in Section 6 achieve higher performance than the default one in a total of six different network scenarios for all considered traffic conditions.

## 8. Conclusions

Configuring the ZigBee stack to work optimally in an HA environment is not a trivial task. The ZigBee base specification and the ZigBee HA profile apply restrictions on the stack configuration. However, no indications for performance-aware configuration are included in these documents. Furthermore, a holistic performance evaluation of the ZigBee protocol stack, the influence of settings from each layer on the performance prevailing, is not available in the literature.

Several WHAN scenarios with different topologies, traffic loads and node roles were defined and implemented in a real testbed to carry out experiments with a large variety of communication protocol stack configurations. The evaluation of these scenarios has shown that the mechanisms and the configuration parameters from different layers of the ZigBee specification can have a significant effect on the overall network performance.

At the MAC layer, the measurements that are carried out with different maxima of MAC layer retransmissions show the importance of one-hop reliability for overall network performance. The findings show that increasing this limit greatly improves the performance, especially as the traffic scale increases. Surpassing the limitations defined by ZigBee for the allowed amount of retransmissions may even lead to better results, although, eventually, a point is reached where performance saturates or even degrades due to congestion. The evaluations also showed that there is an important cross layer effect between the maximum amount of MAC layer retransmissions and the behavior of the link break detection mechanism of the NWK layer. A higher LDR, obtained by increasing the allowed number of MAC layer retransmissions, reduces the amount of route repairs, which are initiated after a data frame transmission failure. As a consequence, control message overhead and congestion decrease. Therefore, we suggest to increase the default setting of maximum MAC layer transmissions and its allowed value range.

For the routing metric as part of the NWK layer routing mechanism, the ZigBee specification leaves the implementation of a link cost algorithm to the user. It has been shown that the metric of a default ZigBee stack, as implemented in the widely used BeeStack, or constant link costs (HC metric) do not result in the best performance. As the specification suggests, a mapping from LQI to LDR is recommendable and can lead to a noticeable improvement in the performance. The PLF metric achieves this by using a medium-level granularity mapping that provides sufficient link quality-awareness and, at the same time, a good path diversity. The latter is required to avoid overutilization of a few high quality routes, as the Path-DR metric does.

While the ZigBee specification does not foresee an alternative RTO algorithm for the end-to-end reliability mechanism, we could show that the overall PDR can be increased by using certain alternatives, at the cost of slightly higher delays and less energy efficiency. As a balanced alternative to the default algorithm, the cross layer algorithm presented is a simple to implement and effective solution.

The evaluations show that PDR, delay and energy efficiency can be improved significantly by altering the default stack configuration or by implementing alternative mechanisms at each layer. Two recommended stack configurations were derived from the results obtained from the evaluations of the mechanisms and parameter settings of the ZigBee layers. The Recommended-Compliant configuration of the ZigBee stack defines a higher number of MAC layer retries and uses the PLF routing metric. This configuration provided an improvement of up to 31.5% in terms of PDR, a delay drop of up to 60.4% and an improvement of the energy efficiency of up to 46.5% for specific traffic conditions compared to the default ZigBee configuration for the investigated scenarios. Finally, the Recommended-Unrestricted configuration requires the modification of the maximum number of MAC layer retransmissions and a modification of the APL layer RTO algorithm. By resorting to the PLF as the routing metric, allowing up to 11 MAC layer retries and applying the cross layer RTO algorithm, a PDR improvement of up to 33.6%, a delay reduction of up to 66.6% and an improvement of energy efficiency of up to 48.7% were achieved compared to the default ZigBee configuration for the investigated scenarios. It could be shown that the two recommended stack configurations also outperform the default settings in three different network topologies for two different transmit power settings. In summary, by tuning a subset of the available ZigBee settings, an important performance improvement is achievable, and the addition of alternative settings and mechanisms may further increase it.

Based on the findings of this paper, we recommend the ZigBee Alliance and the IEEE 802.15 Working Group to reconsider the default configuration and/or relax the allowed range of settings for the investigated parameters and mechanisms. Our findings also provide useful guidelines for the configuration and design of non-ZigBee, low-power, wireless networks, which are, however, based on IEEE 802.15.4.

## Figures and Tables

**Table 1.** Key performance feature comparison of different wireless home automation network (WHAN) technologies. BLE, Bluetooth Low Energy.

	ZigBee	Z-Wave	BLE	Wi-Fi *^a^*
RF Band (MHz)	868/915/2400	868/908/2400	2400	2400
Bitrate (kbps)	20/40/250	9.6/40/200	1000	≤ 54 × 10^3^
Receiver sensitivity (dBm)	≤ −85 at 2.4 GHz ≤ –92 at 868/915 MHz	−101 (at 40 kbps)	≤ −70 (required) −87 to −93 (typical)	−68
Max. msg.size (bytes)	127	64	47	2346
Hop limit	30/10 (mesh/tree)	4	1	1

^a^ Referring to the IEEE 802.11g standard when used in infrastructure mode.

**Table 2.** The retransmission timeout (RTO) algorithms investigated and their main aspects. BEB, Binary Exponential Backoff; CL, cross layer; RTT, round-trip time.

Algorithm	Initial RTO	Backoff method

ZigBee	1.5 s	-
ZigBee + Backoff	1.5 s	BEB
Constrained Application Protocol (CoAP)	[2 *s*, 3 *s*]	BEB
CL	4 × *RTT_RE_*	BEB
Request For Comments (RFC) 6298	1.5 s	BEB

**Table 3.** Overview of possible events for the three node roles.

Role	Aperiodic events	Periodic events
A	Yes	No
B	Yes	Yes
C	Yes	No

**Table 4.** Average number of tries that are performed by the MAC layer to transmit one unicast data frame for different metrics and traffic scenarios.

Metric	Low Traffic	Medium Traffic	High Traffic
BeeStack	1.32	1.44	1.63
HC	1.30	1.45	1.64
PLF	1.22	1.38	1.59
Path-DR	1.28	1.47	1.67

**Table 5.** Performance improvements achieved by the recommended stack configurations over the default ZigBee stack configuration for different topologies (default transmission power, high traffic scale).

	Basic		Dumbbell		Square	

	Compl.	Unrestr.	Compl.	Unrestr.	Compl.	Unrestr.
PDR	31.5%	33.6%	2.7%	2.8%	19.2%	24.8%
Delay	−55.3%	−61.9%	−32.4%	−32%	−39%	−47.8%
Energy Efficiency	+21.4%	+28%	+23.9%	+22.8%	+16.4%	+19.5%

**Table 6.** Performance improvements achieved by the recommended stack configurations over the default ZigBee stack configuration for different topologies (increased transmission power, high traffic scale).

	Basic		Dumbbell		Square	

	Compl.	Unrestr.	Compl.	Unrestr.	Compl.	Unrestr.
PDR	+0.8%	+1%	+0.4%	+0.3%	+0.2%	+0.1%
Delay	−47.7%	−49.1%	−24.7%	−21.8%	−27%	−30%
Energy Efficiency	+37%	+36.5%	+12.2%	+12.5%	+11.1%	+15.7%
